# Utilizing the Electronic Health Record to Evaluate Lipoprotein(a) Testing Within a Large Regional Health System

**DOI:** 10.1016/j.jacadv.2024.101441

**Published:** 2024-12-05

**Authors:** Kamil F. Faridi, Qifan Wu, Chungsoo Kim, Erica S. Spatz, Nihar R. Desai, Harlan M. Krumholz, Yuan Lu

**Affiliations:** a Section of Cardiovascular Medicine, Department of Medicine, Yale School of Medicine, New Haven, Connecticut, USA; b Center for Outcomes Research and Evaluation, Yale New Haven Health, New Haven, Connecticut, USA

**Keywords:** lipids, lipoprotein(a), prevention, testing

**What is the clinical question being addressed?**

How is lipoprotein(a) being tested in real-world practice?

**What is the main finding?**

Lipoprotein(a) testing is rare in current clinical practice, particularly among outpatient primary care providers.

Elevated lipoprotein(a) [Lp(a)] is an established risk factor for atherosclerotic cardiovascular disease (ASCVD), affecting approximately 20% of adults.^1^ Recognizing its significance, multiple guidelines including the recently updated scientific statement by the U.S. National Lipid Association, now recommend Lp(a) testing be performed once in all adults.^2^, ^3^, ^4^ However, there are limited data on Lp(a) testing in real-world clinical practice. This study analyzed electronic health record (EHR) data from a large, multi-hospital health care system to characterize patterns of care and evaluate the potential for large-scale digital population risk surveillance.

## Methods

We conducted a retrospective study using EHR data within the Yale-New Haven Health System (YNHHS). YNHHS is Connecticut’s largest health care system, comprising 5 hospitals and an extensive outpatient network. All YNHHS hospitals and clinics use a secure, centralized EHR. The Yale University Institutional Review Board exempted this study and waived informed consent.

Adults aged ≥18 years with an outpatient encounter from January 2013 to December 2021 were included. To reliably capture comorbidities in EHR data, we identified patients who underwent Lp(a) testing with data available for at least 90 days prior to Lp(a) assessment. In our data set, the Lp(a) test was recorded on the same date as the clinical encounter date on which it was ordered. For patients with multiple Lp(a) tests, we included only the first Lp(a) test in the analysis. For patients without Lp(a) testing, we included those with a lipid panel and ≥90 days of data preceding the first available test. We compared demographics, comorbidities, and medications for patients with and without Lp(a) testing. We also assessed patients with elevated and normal Lp(a) levels, provider specialty, and outpatient versus inpatient Lp(a) testing. All analyses were conducted using R software (version 4.2.3) (R Foundation, Vienna, Austria).

## Results

A total of 164,072 patients were included, of whom 1,477 (0.9%) had Lp(a) assessed. Comorbidities at the time of lipid assessment were determined from a median of 8 (IQR: 4-17) clinical encounters with a median of 3.5 (IQR: 2.0-5.3) years of data available prior to testing. Patients without Lp(a) were more likely to be women, Black, or Hispanic/Latino. They also had lower prevalence of ASCVD and lower usage of lipid-lowering therapies (standard difference ≥10% for all), with similar blood cholesterol values (Table 1).

**Table 1:** Characteristics of Patients With and Without Lp(a) Testing

	Lp(a) Assessed (n = 1,477)	Lp(a) Not Assessed (n = 162,595)	Standardized Difference
Age, y	56 ± 14	56 ± 18	4.4
Female	692 (46.9)	89,820 (55.2)	16.8
Race			
Non-Hispanic White	1,062 (71.9)	109,708 (67.5)	13.7
Non-Hispanic Black	157 (10.6)	20,992 (12.9)	
Non-Hispanic Asian	34 (2.3)	4,350 (2.7)	
Hispanic	122 (8.3)	18,170 (11.2)	
Other/unknown	102 (6.9)	9,375 (5.8)	
Insurance			
Private	1,048 (71.1)	96,534 (59.4)	25.1
Public	348 (23.6)	54,077 (33.3)	
Self-pay	19 (1.3)	2,177 (1.3)	
Other/unknown	60 (4.1)	9,807 (6.0)	
BMI, kg/m^2^	28.8 ± 6.1	29.4 ± 6.9	8.1
Hypertension	460 (31.1)	41,537 (25.5)	12.4
Diabetes mellitus	152 (10.3)	15,229 (9.4)	3.1
CKD	22 (1.5)	3,524 (2.2)	5.1
CAD	364 (24.6)	12,803 (7.9)	46.7
Stroke	241 (16.3)	5,398 (3.3)	44.8
PAD	13 (0.9)	724 (0.4)	5.4
Any ASCVD	569 (38.5)	17,155 (10.6)	68.7
Premature ASCVD	260 (17.6)	3,127 (1.9)	54.8
Active smoker	120 (8.3)	17,061 (10.9)	10.4
No prior history of ASCVD	n = 908	n = 145,440	
10-y risk, %	9.5 ± 17.7	13.9 ± 22.6	21.3
<5%	534 ± 58.8	71,984 ± 49.5	33.1
5.0-7.4%	90 ± 9.9	11,419 ± 7.9	
7.5-19.9%	172 ± 18.9	27,819 ± 19.1	
≥20%	104 ± 11.5	27,777 ± 19.1	
Lipids at the time of Lp(a) assessment, mg/dL			
Total cholesterol	174 (142-208)	178 (151-207)	4.9
Non-HDL-C	119 (90-150)	122 (97-149)	2.1
LDL-C	97 (71-126)	101 (78-125)	3.7
HDL-C	51 (41-63)	52 (42-65)	7.5
Triglycerides	101 (72-147)	100 (72-145)	5.2
LDL-C >190 mg/dL	47 (3.2)	2,447 (1.5)	11.1
No prior history of ASCVD, medication use	n = 908	n = 145,440	
Statin	337 (37.1)	29,515 (20.3)	37.8
Ezetimibe	44 (4.8)	1,175 (0.8)	24.5
PCSK9i	9 (1.0)	100 (0.1)	12.7
Prior history of ASCVD, medication use	n = 569	n = 17,155	
Statin	363 (63.8)	8809 (51.3)	25.4
Ezetimibe	65 (11.4)	888 (5.2)	22.8
PCSK9i	22 (3.9)	146 (0.9)	20.0

ASCVD = atherosclerotic cardiovascular disease; BMI = body mass index; CAD = coronary artery disease; CKD = chronic kidney disease; HDL-C = high-density lipoprotein cholesterol; LDL-C = low-density lipoprotein cholesterol; Lp(a) = lipoprotein(a); PAD = peripheral artery disease; PCSK9i = proprotein convertase subtilisin/kexin type 9 inhibitor; SD = standard deviation.

Values are mean ± SD, n (%), or median (25th-75th percentile). Standardized differences >10 were considered statistically significant.

Among all patients with Lp(a) testing, 74.6% were outpatient tests and 25.4% were performed during a hospitalization. Among patients with outpatient Lp(a) testing, 16.5% were ordered by primary care, 49.0% by a cardiologist, and 3.7% by an endocrinologist. Among all outpatient health care providers, 4.6% of primary care providers and 14.1% of cardiologists ordered at least 1 Lp(a) test. Among patients with inpatient testing, 56.5% presented with acute ischemic stroke and 17.2% presented with acute myocardial infarction. Patients who were Black and those with ASCVD had higher prevalence of elevated Lp(a) (Table 2). Among patients with only a standard outpatient lipid panel, 53.0% were ordered by a primary care provider, 10.3% by a cardiologist, and 2.0% by an endocrinologist.

**Table 2:** Characteristics of Patients With Lipoprotein(a) Testing Performed

	Lp(a) ≥50 mg/dL or ≥125 nmol/L (n = 435)	Lp(a) <50 mg/dL or <125 nmol/L (n = 1,042)	Standardized Difference
Age, y	56 ± 13	55 ± 14	4.1
Female	214 (49.2)	478 (45.9)	6.7
Race			
Non-Hispanic White	281 (64.6)	781 (75.0)	35.2
Non-Hispanic Black	80 (18.4)	77 (7.4)	
Non-Hispanic Asian	6 (1.4)	28 (2.7)	
Hispanic	34 (7.8)	88 (8.4)	
Other/unknown	34 (7.8)	68 (6.5)	
Insurance			
Private	313 (72.0)	735 (70.5)	10.9
Public	93 (21.4)	257 (24.7)	
Self-pay	6 (1.4)	13 (1.2)	
Other/unknown	23 (5.3)	37 (3.6)	
BMI, kg/m^2^	29.1 ± 6.6	28.7 ± 5.8	5.3
Hypertension	143 (32.9)	317 (30.4)	5.3
Diabetes mellitus	50 (11.5)	102 (9.8)	5.5
CKD	14 (3.2)	8 (0.8)	17.6
CAD	119 (27.4)	245 (23.5)	8.8
Stroke	84 (19.3)	157 (15.1)	11.3
PAD	4 (0.9)	9 (0.9)	0.6
Any ASCVD	185 (42.5)	384 (36.9)	11.6
Premature ASCVD	86 (19.8)	174 (16.7)	8.0
Active smoking	30 (7.1)	90 (8.8)	12.7
No prior history of ASCVD	n = 250	n = 658	
10-y risk, %	10.0 ± 18.8	9.4 ± 17.3	3.2
<5%	147 ± 58.8	387 ± 58.8	7.7
5.0-7.4%	25 ± 10.0	65 ± 9.9	
7.5-19.9%	51 ± 20.4	121 ± 18.4	
20%	25 ± 10.0	79 ± 12.0	
Lipids at the time of Lp(a) assessment, mg/dL			
Total cholesterol	179 (143-214)	174 (140-206)	9.3
Non-HDL-C	121 (90-154)	118 (90-149)	4.6
LDL-C	100 (72-130)	96 (70-125)	8.9
HDL-C	52 (41-65)	50 (40-62)	13.6
Triglycerides	98 (71-134)	103 (73-153)	14.6
LDL-C >190 mg/dL	16 (3.7)	31 (3.0)	3.9
Lp(a) values			
nmol/L (n = 1393)	203 (162-301)	24 (10-50)	265.5
mg/dL (n = 84)	94 (63-136)	12 (4-28)	214.5
No prior history of ASCVD, medication use	n = 250	n = 658	
Statin	107 (42.8)	230 (35.0)	16.1
Ezetimibe	17 (6.8)	27 (4.1)	11.9
PCSK9i	4 (1.6)	5 (0.8)	7.8
Prior history of ASCVD, medication use	n = 185	n = 384	
Statin	123 (66.5)	240 (62.5)	8.3
Ezetimibe	23 (12.4)	42 (10.9)	4.7
PCSK9i	11 (5.9)	11 (2.9)	15.1

Abbreviations as in Table 1.

Values are mean ± SD, n (%), or median (25th-75th percentile). Standardized differences >10 were considered statistically significant.

## Discussion

In this study of more than 160,000 patients, we demonstrated that the frequency of Lp(a) testing in real-world U.S. practice is low. Certain groups such as women, Black, or Hispanic/Latino patients are less likely to get tested. Consistent with prior evidence across racial groups,^1^ Black patients were more likely to have high Lp(a) levels when assessed, even though we found there were fewer Black patients among those who received testing. Cardiologists performed half of all outpatient testing, though only 14% of cardiologists and 5% of primary care providers caring for patients had an outpatient Lp(a) test ordered.

Our findings indicate the need for greater awareness and guideline integration to increase Lp(a) testing, particularly within primary care. Notably, we found that certain groups were more likely to be tested (eg, patients who were White, had LDL-C >190 mg/dL, or lower 10-year ASCVD risk among primary prevention patients). These differences could be due to the specific practice patterns and unique patient populations of the relatively few providers who tested for Lp(a) (eg, lipid experts who may receive referrals). However, most guidelines now recommend testing regardless of these other factors. Expansion of testing will therefore be needed to reduce disparities in risk assessment and management, particularly for important demographic groups such as women and racial/ethnic minorities. Furthermore, these findings underscore the importance of using EHR data to develop metrics for quality improvement in public health. Physician education on current guidelines along with incorporation of Lp(a) testing as a standardized health metric within health systems could help improve testing rates.

This study has several limitations. Though the patient population has characteristics similar to the general U.S. population, findings may differ from other health systems. This study was also limited to structured variables within the EHR. Though guidelines currently recommend a single Lp(a) test irrespective of setting, serum levels evaluated in the inpatient setting may not reflect chronic Lp(a) levels.^5^ Lastly, future studies should evaluate larger populations across multiple health centers and geographic regions, and implementation strategies should assess how to increase testing by health care providers.

In conclusion, we demonstrated that Lp(a) testing is rare in current clinical practice, particularly among outpatient primary care providers. To meet current recommendations for Lp(a) risk evaluation in all adults, substantial efforts will be needed to rapidly expand testing. EHR-based surveillance could help facilitate this transition and assess the adequacy of Lp(a) testing across health systems.

## Funding support and author disclosures

Dr Faridi has received research funding from the 10.13039/100000050NIH/NHLBI (K23HL161424), outside the scope of the current work. Dr Lu received research support from the Sentara Research Foundation, Novartis, the 10.13039/100000050National Heart, Lung, and Blood Institute (grant Nos. R01HL169954, R01HL169171), and the Patient-Centered Outcomes Research Institute (No. HM-2022C2-28354) through Yale University. In the past 3 years, Dr Krumholz has received expenses and/or personal fees from UnitedHealth, Element Science, Aetna, Reality Labs, Tesseract/4Catalyst, F-Prime, the Siegfried and Jensen Law Firm, Arnold and Porter Law Firm, and Martin/Baughman Law Firm; he is a cofounder of Refactor Health and HugoHealth; and he is associated with contracts, through Yale New Haven Hospital, from the Centers for Medicare & Medicaid Services and through Yale University from Johnson & Johnson. Dr Spatz has received funding from the Centers for Disease Control and Prevention (20042801-Sub01), the National Heart, Lung, and Blood Institute (R01HL151240), and the Patient Centered Outcomes Research Institute (HM-2022C2-28354). All other authors have reported that they have no relationships relevant to the contents of this paper to disclose.
